# Grazing exclusion had greater effects than nitrogen addition on soil and plant community in a desert steppe, Northwest of China

**DOI:** 10.1186/s12870-021-03400-z

**Published:** 2022-02-03

**Authors:** Mengru Li, Lilong Wang, Junjun Li, Zhenling Peng, Liang Wang, Xinfang Zhang, Shijian Xu

**Affiliations:** 1 School of Life Sciences, Lanzhou University, No. 222, Southern Tianshui Road, Lanzhou, 730000 China; 2 Northwest Institute of Eco-Environment and Resources, CAS, Lanzhou, 730000 China; 3 Administration of Anxi Extra-arid Desert National Nature Reserve, Guazhou, 736100 China

**Keywords:** Nitrogen addition, Exclosure, Desert steppe, Plant community, Nutrient

## Abstract

**Background:**

The impacts of increasing nitrogen (N) deposition and overgrazing on terrestrial ecosystems have been continuously hot issues. Grazing exclusion, aimed at restoration of grassland ecosystem function and service, has been extensively applied, and considered a rapid and effective vegetation restoration method. However, the synthetic effects of exclosure and N deposition on plant and community characteristics have rarely been studied. Here, a 4-year field experiment of N addition and exclusion treatment had been conducted in the desert steppe dominated by *Alhagi sparsifolia* and *Lycium ruthenicum* in northwest of China, and the responses of soil characteristics, plant nutrition and plant community to the treatments had been analyzed.

**Results:**

The grazing exclusion significantly increased total N concentration in the surface soil (0-20 cm), and increased plant height, coverage (*P* < 0.05) and aboveground biomass. Specifically, *A. sparsifolia* recovered faster both in individual and community levels than *L. ruthenicum* did after exclusion. There was no difference in response to N addition gradients between the two plants.

**Conclusions:**

Our findings suggest that it is exclusion rather than N addition that has greater impacts on soil properties and plant community in desert steppe. Present N deposition level has no effect on plant community of desert steppe based on short-term experimental treatments.

**Supplementary Information:**

The online version contains supplementary material available at 10.1186/s12870-021-03400-z.

## Background

The increasing aerial nitrogen (N) deposition derived from the intensification of both agricultural and industrial activities is affecting the ecosystems worldwide. As Moore had pointed out that it is too much of a good thing [1]**, t**he increased N deposition can change the nutrient and moisture status of the soil [2, 3], alter nutrient cycle of ecosystems [4], facilitate the growth of nitrophilic plants [5, 6], deteriorate biological diversity [7, 8], even alter community structure, composition and function of terrestrial ecosystems [5, 7, 9–11]. These effects are more likely to be found in some N-limited terrestrial ecosystems such as vegetation in arid environment [9, 12–14].

One of the important causes of the above consequences is that the increased available N changes the way plants use and recycle nutrients, such as nutrient allocation patterns, foliar chemistry and nutrient resorption [5, 15]. Nutrient resorption, a process of nutrient transferring from senescent tissues to mature tissues [16], is one of the key nutrient conservation strategies, therefore, has considerable adaptive and functional significance [5], specifically for plants in oligotrophic environment. Generally, plant nutrient resorption is associated with plant functional forms (for examples, legume and non-legume) [17, 18], and strongly influenced by nutrient availability [19]. Symbiotic N fixation broadens potential N resources and generally increases N absorption, which results in stable N concentration and N resorption in legumes [14, 17, 18]. Nutrient resorption patterns can also be altered by soil nutrient availability, although the divergent results have been found based on either inter- or intra-species studies [5, 16, 20]. Given the increasing N deposition scenarios, studies demonstrated that N addition resulted in higher availability of soil inorganic N [21], thus increased the leaf N and phosphorous (P) concentrations [22, 23], and decreased the nutrient resorption efficiency [20, 24]. However, even in the same experiment, species-scale nutrient resorption was different in response to N addition. For example, Lü et al. found that only half of the measured species reduced both N and P resorption efficiency in response to increased N inputs in a temperate steppe [5]. The diverse results indicate that more manipulate experiments are needed for better understanding the regulating mechanisms of N enrichment on ecosystem productivity and predicting plant community composition in a nutritionally restricted ecosystem such as desert steppe .

Arid area, accounting for 41% of the earth's land and supporting 38% of the population, is one of the most sensitive ecosystems responding to global change [25, 26]. Overgrazing have led to severe soil degradation, decrease in vegetation coverage [27, 28], ultimately, lowered the productivity [29]. These consequences have been more common in northwestern China in the past decades [30]. As one of the most extensive approaches, exclusion has been implemented for self-recovery of the overgrazed desert since 2004 in China [29, 31]. Generally, grazing exclusion can effectively facilitate soil fertility [32], increase plant N concentration [33, 34], vegetation coverage and plant composition [35, 36], therefore, increase the biomass accumulation of plant communities and facilitate vegetation restoration [37, 38]. However, the inconsistent results were also obtained [39–41]. Therefore, the knowledge is critical for a comprehensive understanding of the effects of grazing exclusion and N addition on soil properties, plant nutrition and vegetation recovery in this area. Here, 4-year N addition and grazing exclusion experiments were conducted in the desert steppe consisting of a legume and a non-legume species at western Hexi Corridor in China. We hypothesized that (1) exclusion would have a better protective effect on legume, while no or a few effects on non-legume because of the preference of livestock for legume; (2) N addition would increase the nutrient concentrations in soil and plant tissues, promote plant growth, improve aboveground biomass, specifically for non-legume living in low N environment such as desert steppe. To assess the above hypotheses, we determined nutrient status (N and P concentrations) of plant tissues and soil, and investigated plant cover, height and plant aboveground biomass. Our aim addresses to (1) discover the responses of the soil and plant nutrient characteristics to N deposition and exclusion in the desert steppe; (2) reveal the synergistic effect of N deposition and exclusion on plant community structure.

## Methods

### Study site

**Fig. 1:**
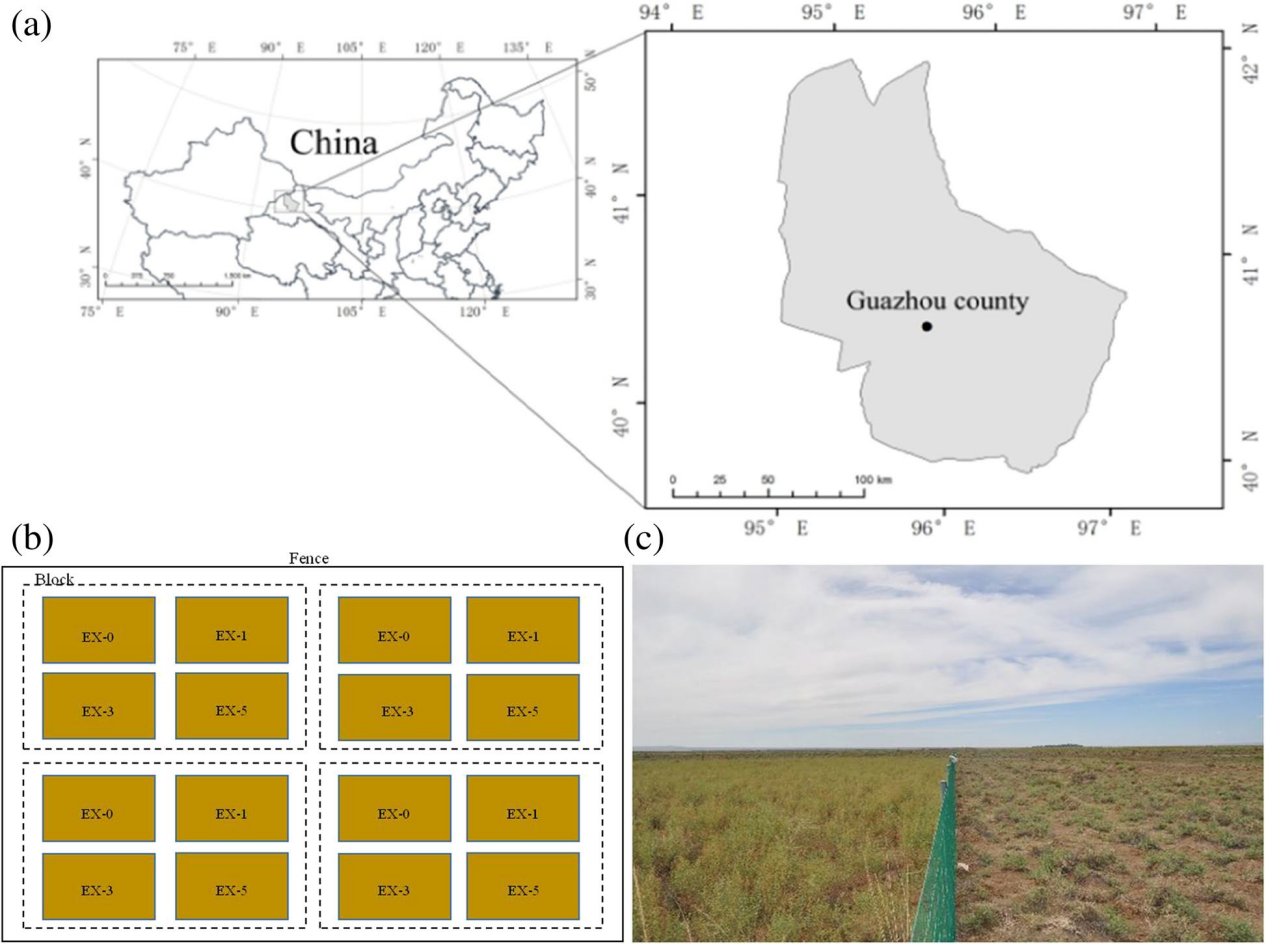
Location of the study site (**a**) (from https://www.webmap.cn) and the experimental design (**b**), in which EX-0, EX-1, EX-3, EX-5 and FG-0 represent the amount of added nitrogen, 0, 1, 3, and 5 g N m^-2^ a^-1^ under fence treatments, respectively. Subfigure (**c**) shows the vegetation in enclosure (left of fence) and in grazing (right of fence) blocks

This study was conducted in a desert steppe ecosystem (aridity index < 0.02) located at the experimental area of Anxi Extra-arid Desert National Nature Reserve (40°16' 56.90” N, 96°11' 52.70” E, 1325 m a.s.l) at western Hexi Corridor in Gansu province, China (Fig. 1), with a mean annual temperature of 8.7°C, a mean annual precipitation of 45 mm, and an annual evaporation of 3000 mm [42]. The harsh environment restricted human activities to traditional uses, grazing with minimal agriculture. Based on investigation when the blocks set up, the vegetation is dominated by *Lycium ruthenicum* Murr. and *Alhagi sparsifolia* Shap*.* accompanied by *Achnatherum splendens* (Trin.) Nevski and *Scorzonera mongolica* Maxim (Table Sup. 1). *A. sparsifolia* is a perennial semi-shrub belonging to Leguminosae with a good feeding value [43], while *L. ruthenicum* is a representative perennial shrub belonging to Solanaceae. Both plants possess well-developed root system, drought tolerance and salinity tolerance, which make them dominant vegetation in sandy environment. Dominant grazing animal in the region is sheep. The grazing intensity (with a stocking rate of 2.43 sheep ha^-1^ year^-1^) in the rangeland is high [42].

### Experimental design

Four enclosed blocks were set up and then N addition and enclosing started in 2014. Each block contains 4 of 10 m × 10 m plots. The blocks and plots were separated by buffer zones of 5 m gaps. N addition was conducted in the form of NH_4_NO_3_ under gradients of 0, 1, 3, and 5 g N m^-2^ a^-1^, hereafter, the enclosed plots were denoted as EX-0, EX-1, EX-3 and EX-5 (Fig. 1). Meanwhile, four plots not less than 15m apart from each other were established in the grazing area as controls, named as FG-0. The exclusion and control sites were located in the same homogeneous ecological units. Half of the fertilizer was dissolved in 10 L water and applied to the plots with a portable sprayer in a rainy or cloudy day in end of May and July, respectively. For EX-0 and FG-0 plots, only 10 L of water was sprayed. The highest N addition level (EX-5, 5 g N m^-2^ a^-1^) was equivalent to the current maximum N deposition at the northern China plain [44].

### Sampling and chemical analysis

Representative sun-exposed, full-expanded mature leaves of the dominant plant species, *L. ruthenicum* and *A. sparsifolia*, were sampled not less than 100 g from not less than five individuals in each plot in middle of July (the peak growing period) and in September (recently senesced, often yellow) in 2018 [13], respectively. The samples were mixed thoroughly in a paper envelope and taken back to the laboratory. After oven-dried at 80°C to a constant weight, the samples were ground using a ball mill (MM 400; Retsch, Haan, Germany) and sieved through a 0.25 mm mesh screen for chemical analysis [45].

The soil samples were collected and treated according to the methods of previous study [46]. The triplicate surface layer (0-20cm) soil samples were taken randomly from each plot in July when the plant samples were collected. Fresh soil was placed in an aluminum box and weighed in situ using an electronic balance, and then dried at 105°C for 24 h in the laboratory to determine the soil water content. The remaining air-dried soil samples were sieved through a 0.15 mm sieve to remove roots and litter residue, and then ground into a fine powder using a ball mill. Soil electrical conductivity and pH were measured on 1: 5 soil : water extracts (2220, Spectrum, USA) and 1 : 2.5 with a pH electrode (IQ150, Spectrum, USA), respectively. The available N (NH+ 4-N and NO- 3-N) was determined using a FIAstar 5000 Analyzer (Foss Tecator, Denmark). Total N was measured by an elemental analyzer (FLASHEA 1112 Series CNS Analyzer, Termo, USA). Total P was determined using the ammonium molybdate method after persulfate oxidation.

In the growth peak season (July), three subplots of 3 m×3 m were set in each plot of the enclosed and free grazing areas. The plant height, species and community coverage and plant density were measured and recorded in each subplot. At the same time, the aboveground parts of *A. sparsifolia* and the leaves and current year's branches of *L. ruthenicum* were collected for the calculation of aboveground biomass. The species-sorted samples were oven-dried at 80°C for 48 h, then, the aboveground community biomass was estimated through total dry mass of all living species per subplot averaged over all replicates of each treatment [47].

### Nutrient resorption efficiency calculations

RE of N and P (NRE and PRE, respectively) was calculated for each species and expressed as the following [20]:

$$RE=\left(1-\frac{Nu_{\mathrm{senesced}}}{Nu_{green}}\right)\times 100\%$$

in which Nu_green_ and Nu_senesced_ are N or P concentration of green or senesced leaves (N_green_, P_green,_ N_senesced_, P_senesced_, respectively) based nutrient mass per leaf dry mass, respectively.

### Statistical analysis

Levene's test was used to test for normality of all data before statistical analysis, and the data were log 10 transformed when it was necessary to obtain approximate normality and homogeneity of residuals. The means of leaf N, P concentrations and physicochemical properties of the soil for each N addition rate were separated by using multiple comparison. One-way ANOVA (Duncan test) was used to test the impacts of treatments on leaf N, P concentrations and nutrient RE. The relationships between soil nutrient concentrations, physicochemical properties and plant leaf element concentrations were analyzed by Pearson correlation. The independent samples t-test was used to determine the differences in plant N and P concentrations between *L. ruthenicum* and *A. sparsifolia* under each treatment. All data analysis and mapping were conducted with SPSS version 18.0, Origin 8.0 and ArcGIS 10.2. The significance level was set at *P* = 0.05 for all calculations.

## Results

### Soil physicochemical properties and nutrient characteristics

The higher total N concentration was found in the soil of enclosure than that in the free grazing area (*P* < 0.05) (Table 1). Specifically, exclusion significantly increased soil NH+ 4-N and NO- 3-N concentrations (in EX-0), and decreased the soil pH and soil water content than free grazing did (FG-0) (*P* < 0.05). The N addition rates increased the soil NH+ 4-N concentration (*P* < 0.05) (Table 1), however, demonstrated no significant effect on the soil NO- 3-N and total N concentrations, pH, EC and soil water content in the enclosed sites (*P* > 0.05) (Table 1).

**Table 1:** Nutrient and ion content, and physicochemical properties in surface soil after 4-year nitrogen addition treatment in exclosure and free grazing sites

Site	TN (mg/g)	TP (mg/g)	K (mg/g)	Na (mg/g)	NH+ 4-N (mg/kg)	NO- 3-N (mg/kg)	pH	EC (mS/cm)	SWC (%)
FG-0	**0.89±0.03b**	0.52±0.03	**0.45±0.09b**	15.97±2.34	**5.69±0.31c**	**1.54±0.12b**	**8.15±0.09a**	8.25±0.10	**7.46±0.62a**
EX-0	**1.49±0.04a**	0.43±0.03	**2.57±0.23a**	17.59±2.10	**6.86±0.76c**	**3.02±0.52ab**	**7.47±0.11b**	8.90±1.32	**3.18±0.74b**
EX-1	**1.61±0.09a**	0.46±0.02	**2.84±0.21a**	17.71±2.03	**17.21±0.31b**	**3.41±0.28a**	**7.51±0.06b**	9.25±0.77	**3.21±0.43b**
EX-3	**1.58±0.06a**	0.44±0.04	**2.87±0.23a**	18.90±1.23	**18.74±0.73ab**	**3.03±0.64ab**	**7.27±0.11b**	9.09±0.76	**2.80±0.69b**
EX-5	**1.54±0.03a**	0.48±0.04	**2.89±0.35a**	19.53±0.61	**19.46±0.31a**	**3.62±0.54a**	**7.47±0.10b**	10.60±0.61	**3.58±0.73b**

Note: *FG* free grazing, *EX* exclosure. Data are means ± standard error (*n* = 4), with different letters representing significant differences between treatments (*P <* 0*.*05). EC: electrical conductivity; SWC: soil water content

### Leaf element concentrations and nutrient resorption efficiency

**Fig. 2:**
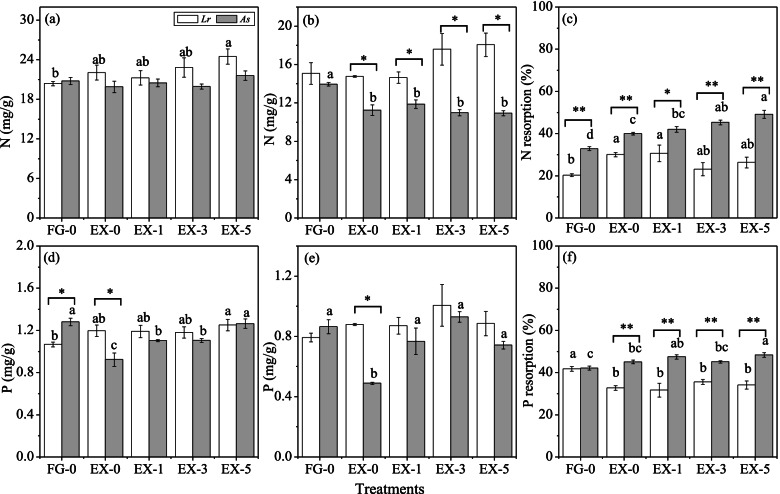
Nitrogen concentrations (N, a, b), phosphorus concentrations (P, d, e) in green (a, d) and senesced leaves (b, e) of *Lycium ruthenicum* (*Lr*) and *Alhagi sparsifolia* (*As*), N (c) and P (f) resorption efficiency of *L. ruthenicum* and *A. sparsifolia* after 4 years nitrogen addition and exclosure (EX)/free grazing (FG) treatments in arid desert steppe. The columns marked with different letters differ significantly between EX and FG, and among additional nitrogen treatments in *L. ruthenicum* and *A. sparsifolia*, respectively (ANOVA Duncan test, *P <* 0*.*05). * and ** represent significant differences between *L. ruthenicum* and *A. sparsifolia* at *P* < 0.05 and *P* < 0.01 level (*t*-test), respectively

Compared with free grazing, the exclusion treatment decreased P_green_ of *A. sparsifolia* (*P* < 0.05) (Fig. 2 d), but showed no significant effect on N_green_ of both plants (Fig. 2 a). However, the exclusion treatment decreased N_senesced_ and P_senesced_ of *A. sparsifolia* (*P* < 0.05), and showed no significant effect on both N_senesced_ and P_senesced_ of *L. ruthenicum* (Fig. 2 b, e). The N addition increased P_green_ and P_senesced_ of *A. sparsifolia* (Fig. 2 d, e). It is worth noting that the N_senesced_ in *A. sparsifolia* was significantly lower than that in *L. ruthenicum* (*P* < 0.05) at each N addition treatment level (Fig. 2 b).

N addition increased NRE and PRE of *A. sparsifolia* (*P* < 0.05) (Fig. 2 c, f), however, no significant change was detected in *L. ruthenicum* (Fig. 2 c, f). The exclusion treatment significantly increased NRE of the two plants (*P* < 0.05) (Fig. 2 c), but showed no effect on PRE of *A. sparsifolia* and decreased PRE of *L. ruthenicum* (*P* < 0.05) (Fig. 2 f) compared with free grazing. *A. sparsifolia* had significantly higher NRE and PRE than *L. ruthenicum* except for PRE in FG-0 (*P* < 0.01) (Fig. 2 c, f).

### Community characteristics after enclosure and nitrogen addition

**Fig. 3:**
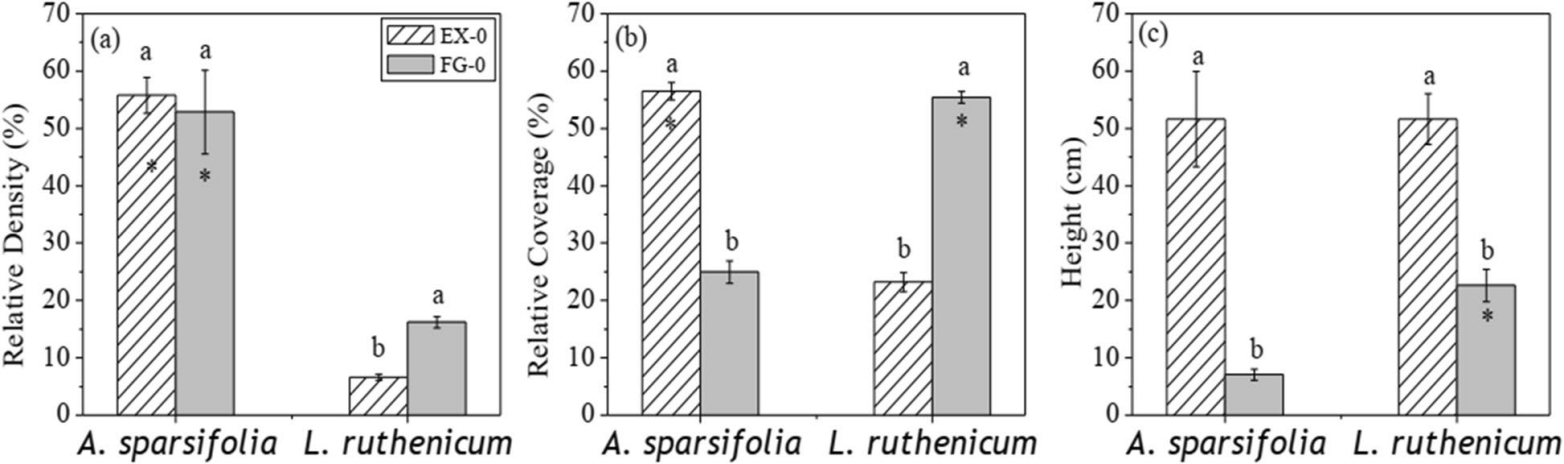
Relative density (%, a), relative coverage (%, b) and height (cm, c) of *Lycium ruthenicum* and *Alhagi sparsifolia* in the enclosure (EX-0) and the free grazing sites (FG-0). Different letters represent significant differences of the same species between EX-0 and FG-0 at *P* < 0.05, while * represents significant differences between *L. ruthenicum* and *A. sparsifolia* under the same treatments at *P* < 0.05 level (t-test)

Exclusion dramatically increased the community Shannon-Wiener Index and the Simpson Index (Table 2), the vegetation coverage and height (Table 3) than those of grazing treatment (*P* < 0.05). Specifically, the exclusion increased annual aboveground biomass (*P* < 0.05) despite of no significant difference in plant density compared with free grazing (Table 3). Exclusion decreased the relative coverage of *L. ruthenicum* but increased the height and the relative coverage of *A. sparsifolia* (Fig. 3). However, 4-year N addition presented no effect on the above traits (Table 2 and Table 3).

**Table 2:** Community diversity index in exclosure and free grazing sites

Site	S	H	D
FG-0	3	1.31±0.06 b	0.55±0.03 b
EX-0	4	1.72±0.16 a	0.68±0.03 a
EX-1	4	1.48±0.12 ab	0.60±0.03 ab
EX-3	3	1.41±0.12 ab	0.60±0.04 ab
EX-5	4	1.60±0.16 ab	0.64±0.04 ab

Note: *FG* free grazing, *EX* exclosure, *S* Species Richness, *H* Shannon-Wiener Index, *D* Simpson Index. Different letters represent significant differences between treatments (*P <* 0*.*05)

**Table 3:** Community characteristics (means ± standard error) in exclosure and free hba

Site	Plant density (individual/m^2^)	Coverage(%)	Height(cm)	Above-ground Biomass(g/m^2^)	*L. ruthenicum* Biomass(g/m^2^)	*A. sparsifolia* Biomass(g/m^2^)
FG-0	8.69±0.62	32.61±1.43 b	13.63±0.99 b	35.85±2.24 b	3.38±0.21	21.80±2.06 b
EX-0	8.41±0.78	56.01±1.53 a	48.33±3.46 a	76.30±1.84 a	3.98±0.25	56.71±1.10 a
EX-1	9.00±1.12	59.33±0.67 a	46.11±1.35 a	73.52±1.56 a	3.81±0.34	55.43±0.80 a
EX-3	8.89±0.44	61.02±2.08 a	48.08±2.01 a	70.87±4.01 a	3.24±0.19	59.43±1.71 a
EX-5	8.48±0.48	58.33±1.86 a	49.11±1.68 a	76.82±2.53 a	3.57±0.29	57.27±0.84 a

Note: *FG* free grazing, *EX* exclosure. Different letters represent significant differences between treatments (*P <* 0*.*05, *n* = 4)

### Relationships between leaf element concentrations and environmental factors

Pearson correlation analysis showed N_green_ concentrations of the two plants were significantly positively correlated with the total N concentration in the soil, but negatively correlated with soil pH (*P* < 0.05) and soil electrical conductivity (*P* < 0.05), while there was no significant correlation between P concentrations in leaves and soil, respectively (Table 4).

**Table 4:** Pearson's correlation coefficients of soil properties with green leaf element concentrations, and nutrient resorption efficiency (n = 4)

	TN	TP	pH	EC	SWC
*L. ruthenicum*					
Leaf N	0.537**	-	-0.423*	-0.484**	-0.075
Leaf P	-	-0.118	-0.387*	-0.382*	-0.076
NRE	0.170	-	0.058	0.100	0.290
PRE	-	-0.090	-0.563*	0.127	-0.076
*A. sparsifolia*					
Leaf N	0.731**	-	-0.659**	-0.373*	-0.028
Leaf P	-	0.108	-0.444*	-0.186	0.145
NRE	0.109	-	-0.179	0.340	-0.031
PRE	-	-0.179	0.059	0.026	-0.217

Note: TN and TP, soil total nitrogen and phosphorus. *EC* electrical conductivity, *SWC* soil water content, *NRE and PRE* nitrogen and phosphorus resorption efficiency. * *P* < 0.05; ** *P* < 0.01

## Discussion

### Grazing exclusion enhanced soil N concentration but had no effect on plant nutrients

Grazing exclusion did not enhance N_green_ of the two plants in this study, which is inconsistent with the previous studies. An & Li (2015) found the leaf N concentration of some species in grazing areas was higher than that in enclosures [34]. They contended that the grazing facilitates the elimination of senescent tissues on the ground and the produce of young tissues with higher nutrient concentration [48]. Therefore, species with high N concentration under the disturbance of grazing is a manifestation of super-compensated growth of plants. On the contrary, Wigley et al. [33] found increased plant N concentration under exclusion treatment which mainly derived from reduction of soil pH and increase in soil nutrients. Given the extreme low soil moisture content (2.8% - 7.46%) in this study, we speculate that soil water availability may be the more important limiting factor for plants nutrient distribution and survival [49], despite of no change in N_green_ in enclosure and no significant correlation between concentrations of N, P in leaves and soil water content. However, more detailed studies would be conducted to show how water and nutrients work together to affect plant survival.

The mass-based measure of nutrient RE may lead to an underestimation of real RE [50], which should be the main reason for the lower NRE and PRE in this study compared with the other studies [13, 16, 20]. However, the underestimation does not affect the difference in nutrient RE among treatments, and the conclusion of this study. Different from N_green_, NRE of *A. sparsifolia* increased significantly after grazing exclusion, which should be the consequence of growth dilution of N concentration due to rapid growth after grazing exclusion [51, 52]. As a manifestation of super-compensated, the enhanced P_green_ resulting from inhibition of growth of *L. ruthenicum* in enclosure accounts for the decreased PRE. The increased total N of soil in the enclosure is consistent with the previous study performed in typical desert [30], and could be attributed to the following account. The increased vegetation coverage and aboveground biomass resulting in exclosure provided good conditions and source for enrichment of total N and organic matter in soil. Therefore, grazing exclusion is an effective method to deal with ecological degradation in arid regions [53].

### Nitrogen addition did not increase the nitrogen concentrations of soil and leaves as expected

Previous studies demonstrate that N addition increased significantly soil N concentrations [54, 55], enhanced N_green_ [22, 31] and reduced foliar NRE [24], which had been attributed to the consequence that the N added to the soil can be quickly converted into available N for plant, and the N level in these plants depends more on soil N resources rather than resorbing from senescent tissues [22, 54, 55]. However, the inconsistent results with the above studies and as we had expected had been found that N addition did not lead to general increase in soil and plant N concentrations. The contrary results should be mainly attributed to the lower dose of N addition employed in this study than that in the other studies. For examples, 20 g N m^-2^ a^-1^ and 10 g N m^-2^ a^-1^ had been employed to simulate N deposition in temperate grassland and temperate forest [55], respectively, while the maximum N addition ratio in this study is 5 g N m^-2^ a^-1^, which is the current largest volume of annual N deposition at the study area [44]. Specifically, the local arid climate, strong evaporation and extreme low soil moisture decrease the mobility and availability of soluble and diffusible substrates and product, severely limit the turnover of soil nutrients, thus affect the availability of nutrients [13]. Therefore, the N addition does not present the expected effect in this study.

The result that nutrient resorption responding to N enrichment was variable at species-scale is consistent with the other study [5]. There is good possibility stemming from the following to interpret the higher N_senesced_ and P_senesced_ and lower NRE and PRE in *L. ruthenicum* rather in *A. sparsifolia* at each treatment level. Firstly, *A. sparsifolia* produces more aboveground biomass each year than *L. ruthenicum* dose. Therefore, more nutrients are needed for *A. sparsifolia* than *L. ruthenicum* under the same growth conditions. In term of survival strategy, *A. sparsifolia* is a fast grower, while *L. ruthenicum* is more conservative ones. Furthermore, livestock prefers legume *A. sparsifolia* rather than *L. ruthenicum*, so the exclosure is more favorable for the growth and biomass accumulation of *A. sparsifolia*, rather than for *L. ruthenicum.* The strong “dilution effect” on N and other nutrients [51, 52] resulting from rapid biomass accumulation in *A. sparsifolia* leads to relative lower nutrient concentration and higher resorption efficiency.

### Exclusion rather than nitrogen addition plays a greater role in maintenance of plant community

The short-term exclusion significantly increased the plant coverage, height and aboveground biomass, and improved the community productivity, which may mainly due to the reduction in food intake by livestock [37, 56, 57]. The fact that the exclusion treatment increased growth of *A. sparsifolia* rather than *L. ruthenicum* should due to the following facts. Since *A. sparsifolia*, a leguminous plant, not *L. ruthenicum,* is preferred by livestock, therefore, the protective effects of livestock exclusion were much greater on *A. sparsifolia* than that on *L. ruthenicum*. On the other hand, the aboveground part of *A. sparsifolia* is annual, which has a faster growth rate than *L. ruthenicum* dose. Therefore, the relative coverage, height and biomass of *A. sparsifolia* increased significantly than *L. ruthenicum* after exclusion treatment. The asymmetric effect of exclusion treatment on the two plants will further lead to the change that *A. sparsifolia* may be the only dominant plant after a longer period of livestock exclusion. The results of N addition experiments overturned our previous hypothesis that the higher N addition will promote rapid growth of non-legume and possibly make it the dominant species. This study also suggests that the current level of N deposition has no effect on structure of plant community in the study area. In addition to the lower level of N addition compared with the other experiments, extremely low soil moisture content should be another key role limiting plant nutrient contents and survival in the arid area [49], directly or indirectly, which, however, needs detailed studies and more data to support. Synthetically, in arid areas with low biodiversity, free grazing rather than N deposition more seriously affected local fragile vegetation. Grazing exclusion can not only increase vegetation coverage and aboveground biomass, but also change the community structure and composition of vegetation, which is far more than the effect of current N deposition level. The impacts of N deposition and exclusion on the vegetation in the desert region still require long-term research. Strategy of moderate grazing rather than absolute isolation should be adopted in process of ecological restoration in arid desert regions.

## Conclusions

In the present study, a 4-year field experiment had been conducted to test the responses of soil properties, plant nutrition and plant community to the N addition and exclusion treatments in the desert steppe in northwest of China. Short-term grazing exclusion significantly increased the soil total N, available N, and increased the plant height, coverage, improved the aboveground biomass. Specifically, legumes *A. sparsifolia* had recovered more than *L. ruthenicum* after exclusion. N addition, however, presented divergent effects on leaf nutrients of the two plants, and no effect on community characteristics. In short, the grazing exclusion, rather than N addition, has greater influence on plant community and surface soil of the desert steppe.

## Supplementary Information

**Additional file 1: Table S1.** The community eigenvalue in EX and FG

## Data Availability

All data generated or analyzed during this study are included in this published article.

## Abbreviations

N: Nitrogen
P: Phosphorus
NRE, PRE: Nutrient resorption efficiency of nitrogen, or phosphorous
Ex: exclosure
FG: Free grazing
