# Supplementary material for: Grazing exclusion had greater effects than nitrogen addition on soil and plant community in a desert steppe, Northwest of China
Source: BMC Plant Biol. 2022 Feb 3;22:60. doi: 10.1186/s12870-021-03400-z (PMC8812004; doi:10.1186/s12870-021-03400-z)
Supplement: Supplementary file 1 — Additional file 1: Table S1. The community eigenvalue in EX and FG [file 12870_2021_3400_MOESM1_ESM.docx]

**Table Sup. 1** The community eigenvalue in EX and FG

| Study sites | Species | Relative Density (%) | Relative Coverage (%) | Relative Frequency (%) | Important Value |
| --- | --- | --- | --- | --- | --- |
| EX | *A. sparsifolia* | 27.03±0.03 b | 63.76±0.04 a | 30.77 | 0.41 |
|  | *L. ruthenicum* | 40.04±0.01 a | 25.1±0.02 b | 30.77 | 0.32 |
|  | *A. splendens* | 20.77±0.11 b | 11.07±0.03 c | 30.77 | 0.21 |
|  | *S. mongolica* | 4.85±0.02 c | / | 5.77 | 0.04 |
| FG | *A. sparsifolia* | 20.79±0.06 b | 17.01±0.02 bc | 30.77 | 0.23 |
|  | *L. ruthenicum* | 46.12±0.11 a | 49.8±0.01 a | 30.77 | 0.42 |
|  | *A. splendens* | 3.68±0.01 c | 10.76±0.01 c | 30.77 | 0.15 |

Note: FG: free grazing; EX: exclosure. Different letters represent significant differences within each column (*P <* 0*.*05).
